# 血脂水平与肺癌风险及预后相关性的研究进展

**DOI:** 10.3779/j.issn.1009-3419.2020.102.36

**Published:** 2020-09-20

**Authors:** 娅 董, 浩澄 王, 东凤 单, 壮 于

**Affiliations:** 266003 青岛，青岛大学附属医院肿瘤科 Department of Oncology, The Affiliated Hospital of Qingdao University, Qingdao 266003, China

**Keywords:** 肺肿瘤, 血脂, 预后, Lung neoplasms, Lipid, Prognosis

## Abstract

近年来，肺癌成为导致癌症相关死亡的主要原因。越来越多证据表明，许多脂类和脂类类似物是肿瘤发生的关键调节因子，吸烟、饮食及肥胖等影响血脂水平的因素可能与癌症的风险相关。目前随着脂质与肿瘤发生过程关系的研究逐渐深入，探索血脂与肺癌风险及预后相关性已成为研究的热点。本文就血脂水平与肺癌发病风险、血脂水平与肺癌患者预后相关性及调整血脂药物与防治肺癌方向的研究进展进行综述。

## Abstract (en)

In recent years, lung cancer has become the leading cause of cancer-related deaths. There is increasing evidence that many lipids and lipid analogs are key regulators of tumorigenesis, and factors that affect blood lipid levels such as smoking, diet, and obesity may be associated with cancer risk. With the deepening of research on the relationship between lipids and tumorigenesis, exploring the correlation between blood lipids and lung cancer risk and prognosis has become a research hotspot. This article reviews the research progress of the relationship between blood lipid levels and the risk of lung cancer, blood lipid levels and the prognosis of lung cancer patients, and the adjustment of blood lipid drugs and the prevention and treatment of lung cancer.

## 1 前言

肺癌是最常见的癌症之一，在全球范围内，肺癌是癌症发病率和死亡率的主要原因，根据国际癌症研究机构发布的2018年全球癌症发病率和死亡率显示，2018年预计有210万例新确诊的肺癌病例和180万例肺癌相关死亡的病例，约占全部癌症相关死亡人数的1/5（18.4%）^[1]^。随着人们对肺癌的逐渐重视和了解，探索各种因素在其发生及发展过程中的作用已成为研究的重点。

脂质在人体内的主要功能是提供能量，并且是维持细胞完整性和许多生物功能（包括细胞生长和分裂）所必需的主要细胞膜成分，它们在正常和恶性组织中起着至关重要的信号传导和提供能源的作用。体内脂质代谢的紊乱可导致某些疾病的发生及发展，血浆中的中性脂肪酸和类脂统称为血脂，血脂与体内脂质系统代谢紊乱和心血管疾病密切相关，因此被认为是监测代谢健康的重要指标。大量的流行病学及实验研究发现，吸烟、饮食及肥胖等影响血脂水平的因素可能与癌症的风险相关，在临床上我们监测血脂水平的主要指标包括总胆固醇（total cholesterol, TC）、甘油三酯（triglyceride, TG）、高密度脂蛋白胆固醇（high density lipoprotein cholesterol, HDL-C）、低密度脂蛋白胆固醇（low density lipoprotein cholesterol, LDL-C）、载脂蛋白A-I（apolipoprotein A-I, ApoA-I）、载脂蛋白B（apolipoprotein B, ApoB）和脂蛋白a。目前随着脂质与肿瘤发生过程关系的研究逐渐深入，而作为直接代表体内脂质水平，并且有着方便、容易获取等特点的血脂水平检测与肺癌发生及预后的关系逐渐成为研究的重点和热点。

## 2 血脂与肺癌发病风险

近年来，流行病学研究已表明吸烟是肺癌的主要危险因素，但是非吸烟肺癌患者发生率的逐年增加为流行病学研究带来了新的方向，研究^[2, 3]^发现肥胖相关指标如饮食、体重指数、血脂、脂蛋白等，也可能与肺癌的风险相关。

### 2.1 血清总胆固醇和甘油三脂与肺癌

胆固醇对细胞膜的完整性，维持细胞膜的流动性和功能，包括信号转导是必不可少的。有前瞻性队列研究^[4]^表明，血清TC水平与癌症总死亡率呈负相关，近年来对于血清胆固醇水平与肺癌发病风险相关性之间的研究也逐渐增加。

为了进一步阐明TC与癌症的关系，Kitahara等^[5]^进行了一项面对韩国人群的前瞻性研究，该研究通过对普通人群长达12年的随访，其中有10, 866人在随访期间确诊肺癌，通过对随访人群的TC水平进行的分析，他们发现具有较高水平血清TC的男性患者比正常者罹患肺癌的风险要小。有学者^[6]^同样认为血清TC浓度与整体癌症风险之间呈负相关，并且发现这种负相关在肺癌和肝癌中尤为明显。一项关于TG与癌症发病率相关性的队列研究^[7]^指出，更高水平的血清TG的人群会增加患肺癌的风险（HR=1.94, 95%CI: 1.47-2.54）。也有研究认为血清TC水平与肺癌发病风险之间并无相关性，Everatt等^[8]^指出虽然肺癌的发病风险与立陶宛男性的体质指数（body mass index, BMI）呈负相关，但是TC水平与肺癌发病率无统计学差异。基于诸多此类研究的结论不同，Lin等^[9]^收集了血脂水平与肺癌风险相关性的队列研究进行*meta*分析，排除临床前癌症对研究结果的影响后共纳入的4项队列研究，他们得出TC与肺癌风险之间呈显著负相关（RR=0.89, 95%CI: 0.83-0.94）。他们同时分析了3项血清TG水平与肺癌发病率相关的队列研究，结果显示两者呈正相关关系。以上研究结果提示胆固醇和脂肪酸代谢可能在肺癌的病因学上呈现不同的、特异的机制，关于TC与TG与肺癌发病风险之间的相关性仍需要大量大规模的前瞻性研究来证实。

### 2.2 血清脂蛋白胆固醇与肺癌

HDL-C和LDL-C是临床上监测脂蛋白胆固醇的主要指标。已有多项研究^[10, 11]^发现，HDL-C与一般人群的癌症风险呈负相关，Ahn^[6]^的研究提出较高的HDL-C水平与降低癌症风险有关，血清HDL-C浓度与肺癌、前列腺癌、肝癌和造血系统癌的发病风险之间呈明显的负相关。近年来的研究表明，HDL-C可能通过其参与胆固醇逆向运输^[12]^、影响细胞周期的进入^[13]^、调节凋亡和炎症反应的作用^[14, 15]^而参与癌症的发生。

近年来有一些对新诊断肺癌的病例对照研究^[16, 17]^表明，肺癌病例中HDL-C水平明显低于以正常人群设置的对照组，这种差异与肺癌的类型与分期无显著相关性。Hao等^[18]^的研究收集了非小细胞肺癌（non-small cell lung cancer, NSCLC）患者的血脂指标，他们发现NSCLC患者普遍存在血脂异常，他们的前瞻性队列研究发现，与低水平HDL-C的人群相比，正常和高水平的HDL-C的人群患NSCLC的风险较低，单因素分析：OR=0.288，95%CI: 0.185-0.448，多因素分析：OR=0.223，95%CI: 0.134-0.407，异常的HDL-C降低与NSCLC的高风险密切相关。Kucharska-Newton等^[19]^对14, 547名社区动脉粥样硬化风险人群进行了前瞻性队列研究，他们分析了基线血浆HDL-C水平与肺癌发病率的关系，结果发现HDL-C水平与肺癌发生率之间存在较弱的关联，他们观察到低HDL-C水平与总样本以及本研究的既往吸烟者（研究开始时已戒烟）中肺癌的发生率相关。一项对于中国男性的前瞻性队列研究^[20]^表明，低水平的LDL-C与肺癌风险增加有关，该研究还提出当同时考虑TC、TG和LDL-C时，异常指标的数量与肺癌风险呈线性关系，具有3种异常指标的人群罹患肺癌的风险比指标全部正常的人群高2倍。

### 2.3 血清载脂蛋白与肺癌

在临床工作中，我们常用的反映患者载脂蛋白水平的生物标志物主要有ApoA-I、ApoB和ApoB/ApoA-I。ApoA是血浆HDL的重要组成部分，ApoA-I是ApoA最常见的一种亚型，而ApoB主要存在于LDL中。已发表的研究^[21, 22]^表明，ApoA-I水平与多种癌症的生存有关。载脂蛋白与肺癌风险之间的关系也有研究。Chandler等^[23]^研究了女性血清ApoA-I、ApoB水平与肺癌、乳腺癌等癌症发病率之间的相关性，他们发现血脂水平与女性患肺癌、结肠癌的总风险相关，低水平的血清ApoB（HR=1.60, 95%CI: 1.03-2.49, *P*=0.006）和高水平的HDL-C（HR=0.59, 95%CI: 0.38-0.93, *P*=0.01）可降低肺癌的发病风险，结果表明一些降低血清ApoB水平或提高HDL-C的生活方式，在预防心脏疾病的同时，还可降低癌症风险。一项面向17, 035名女性和11, 063名男性的一项前瞻性队列研究^[24]^分析了血清血脂水平与癌症风险的相关性，结果显示血清ApoA-I水平与肺癌风险呈负相关（HR=0.88, 95%CI: 0.80-0.97），而高水平的ApoB则增加了肺癌风险（HR=1.08, 95%CI: 0.99-1.18），这与Chandler的研究结果具有一致性。

## 3 血脂与肺癌患者的预后

随着肺癌患病率及发病率的逐年增加，人们对于肺癌的防治给予高度重视。肺癌按其生物学行为的不同，可以将其分为两大类：NSCLC和小细胞肺癌（small cell lung cancer, SCLC），而近年来对于使用血脂指标来预测肺癌预后的研究也逐渐增多。有研究^[25]^表明，胆固醇可能是肺癌的一种预后标志物。官禹等^[26]^共收集了367例初治的NSCLC患者的血清TC、LDL-C、TG指标，他们的研究结果发现血脂高组患者的中位生存期明显高于血脂低组患者，分别为23.4个月和14.6个月，并且血脂异常为NSCLC患者预后的独立因素。Zhang等^[27]^收集了有*EGFR*基因突变的晚期NSCLC患者血清循环胆红素和胆固醇的水平，结果发现TC较高的患者与TC较低的患者相比，死亡风险降低了63%。而与较低HDL-C水平的患者相比，较高HDL-C水平的患者死亡风险降低了46%，而TC可以作为独立的保护因素。Shi等^[28]^发现NSCLC患者的ApoA-I水平明显低于健康对照组，并且治疗血清前ApoA-I水平的降低与NSCLC患者生存期的降低有关。对于血脂与SCLC预后的研究亦有报道，Zhou等^[29]^收集了601例SCLC患者初治时血清LDL-C水平，并把患者分为三组，分别是高、中及低LDL组，结果发现与低LDL组相比，中LDL或高LDL组患者的生存期较低（低LDL、中LDL、高LDL组分别为29.27个月、16.70个月和17.23个月），他们观察到较高的LDL水平是较差的OS的重要独立预后因素，中LDL组与低LDL组相比，死亡风险高1.42倍。

## 4 调节血脂药物在防治肺癌方向的最新进展

调节血脂药物的应用在预防和治疗心血管相关疾病中已有了显著的成效，3-羟基-3-甲基戊二酰辅酶A还原酶抑制剂（他汀类药物）是一种有效的降胆固醇药物，能抑制胆固醇的合成，从而广泛用于预防和治疗心血管疾病。在此背景下一些临床前研究表明，他汀类药物可能通过阻断细胞周期进展^[30]^、诱导细胞凋亡^[31]^、抑制血管生成^[32]^、抑制肿瘤生长和转移^[33]^而具有潜在的抗癌作用。有观察性研究^[34, 35]^表明他汀类药物可能会降低肺癌的发病风险。其中Cardwell^[35]^的研究结果说明肺癌患者使用他汀类药物可以显著降低肺癌特异性死亡率，这项针对大型人群的队列研究结果显示，在肺癌诊断之前接受他汀类药物可使肺癌特异性死亡率最高降低至12%。但是一项共纳入20项临床研究（5项随机对照研究、8项队列研究和7项病例对照研究）的*meta*分析结果显示与不服用他汀类药物的人群相比，服用他汀类药物的人群患肺癌的风险没有实质性的降低。综合以上研究结果，他汀类药物对肺癌可能没有确切的预防作用，有研究^[36]^指出这一结果可能是由于他汀类药物因有选择性的肝吸收和较低的全身可用性，因而对于能否使用他汀类药物来降低肺癌风险有待进一步研究。

关于他汀类药物的使用与肺癌预后之间相关性也有研究。Park等^[37]^发现洛伐他汀可以通过下调RAS蛋白来克服具有*K-Ras*突变的NSCLC细胞中的吉非替尼耐药性，这一结果提示他汀类药物可能与肺癌耐药具有相关性。Lin等^[38]^的研究比较了诊断前接受他汀类药物治疗与未接受他汀类药物治疗的IV期NSCLC患者的生存率，这项研究共纳入了5, 118例患者，结果显示接受他汀类药物治疗组患者的中位生存期比未接受他汀类药物治疗的患者明显延长（7个月*vs* 4个月），他汀类药物的使用提高了肺癌特异性生存率（HR=0.77, 95%CI: 0.73-0.81）。一项关于肺癌和血脂异常患者长期使用他汀类药物的病例对照研究结果^[39]^显示，他汀类药物可以显著降低肺癌死亡率（HR=0.91, 95%CI: 0.86-0.96）。Xia等^[40]^进行了一项关于他汀类药物的使用和肺癌的预后的*meta*分析，他们的结果显示在纳入的观察性研究中（共14篇），他汀类药物可能与降低死亡风险和提高总体生存率有关。以上结果均需要高质量的随机对照研究来证实。

## 5 脂质代谢与肺癌的发生及发展

近年来，越来越多证据表明，脂质代谢改变是恶性肿瘤的一个新特征。许多脂类和脂类类似物是肿瘤发生的关键调节因子，这些证据大多来自对肿瘤细胞的体外研究或通过调节饮食和使用肿瘤细胞异种移植后在动物模型体内进行的研究。脂质可能通过以下机制来参与肺癌的发生及发展。

### 5.1 脂质参与肿瘤细胞信号传导

早期的报道认为，肿瘤细胞吸收和利用外源脂肪酸不仅是为了合成细胞膜以支持其细胞的快速分裂，还生成大量的信号脂质，如磷脂酰肌醇-3, 4, 5-三磷酸（phosphatidylinositol 4-phosphate 3, PIP3）、神经酰胺-1-磷酸（ceramide-1-phosphate, C1P）、溶血磷脂酸（lysobisphosphatidic acid, LPA）、二酰基甘油（diacyl glycerol, DAGs）和血小板激活因子（platelet activating factor, PAF）^[41]^。研究发现这些脂质可增加肿瘤细胞的致病性。PIP3是由磷脂酰肌醇-3-激酶（phosphatidylinositol 3-kinase, PI3K）产生的一种脂质信号传导因子，它通过激活蛋白激酶B/Akt来刺激细胞存活和增殖，这一条通路在促进癌症进展中特别重要^[42]^。C1P是由神经酰胺激酶产生的，有报道^[43]^称C1P可以拮抗神经酰胺的凋亡作用，并通过激活MEK、ERK、PI3K/AKT和JNK等信号通路促进细胞增殖，它通过未知的细胞外G蛋白偶联受体诱导炎症反应并刺激细胞迁移。前列腺素也是另一类重要的脂质信使，由环氧合酶作用产生，支持细胞迁移和肿瘤与宿主的相互作用^[44]^。PAF是一种炎性脂质，它通过PAF受体发挥作用，引发炎症和血小板聚集，它由恶性细胞产生并通过自分泌机制促进侵袭和转移。最近发现的另一个重要的脂质信号传导因子是LPA，它是G蛋白偶联受体家族的典型配体，据报道其可促进多种癌症的侵袭性^[45]^。

### 5.2 脂质筏与癌症

胆固醇在正常细胞和癌细胞中的一个主要功能是它在脂质筏结构域的形成中发挥关键作用，脂质筏是富含胆固醇和鞘磷脂的一种特殊膜结构，它们构成正常细胞和癌细胞中信号转导和运输的组织中心^[46]^，并参与许多生理和病理生理过程，包括细胞凋亡，细胞信号传导等。脂质筏在癌细胞的重要地位体现在它可作为一个平台来分类不同的信号传导过程，并且它们含有许多与癌症相关的信号传导和粘附分子。自分泌或旁分泌的生长因子受体或者配体的信号通路，如胰岛素样生长因子和表皮生长因子通路，可促进肿瘤微环境中癌细胞的增殖和细胞存活，许多生长因子系统的过度表达和过度激活以及生存信号通路对肿瘤的发展至关重要，并且已经证明它们各自下游信号通路的调节取决于它们在脂质筏中的存在^[47]^。临床前模型^[48]^显示，他汀类药物引起的脂质筏耗竭可以减少细胞生长并可使细胞对凋亡刺激的敏感性增加。

## 6 总结

肿瘤脂质代谢异常是近年来备受关注的一个新领域，脂质摄取、储存和代谢的增加发生在各种癌症中，并促进肿瘤细胞的生长^[49]^。有证据^[50]^表明，内源性脂质是肿瘤细胞增殖和细胞死亡机制的重要调节因子，虽然尚未有脂类药物在治疗肺癌方向的研究，但是将脂类药物应用于临床为治疗晚期肿瘤提供新的思路。本综述主要阐述了反映血脂水平的各项指标与肺癌发病风险之间的相关性以及血脂水平与肺癌患者预后之间的相关性，还分析了调整血脂药物与防治肺癌之间的相关性。通过对血脂与肺癌之间各类研究的进展，表明了脂质代谢紊乱与肺癌的发生及发展具有密切的相关性，并成为我们研究肺癌的一个重要的切入方向。
